# Cultural Considerations in Palliative Care Provision: A Scoping Review of Canadian Literature

**DOI:** 10.1089/pmr.2020.0124

**Published:** 2021-05-20

**Authors:** Erynn M. Monette

**Affiliations:** School of Health Studies, Faculty of Health Sciences, University of Western Ontario, London, Ontario, Canada.

**Keywords:** Canada, culture, end-of-life care, palliative care

## Abstract

***Background:*** Palliative care, a division of health care that provides treatment to patients facing terminal or incurable illness, prioritizes maintaining quality of life for the patients it serves. Factors that influence quality of life are highly individualized, encompassing social, economic, or cultural determinants of health. In particular, cultural determinants remain an understudied element of palliative care.

***Objectives:*** The purpose of this article is to identify key concepts and issues arising from offering culturally relevant palliative care by reviewing how the concept of culture has been discussed in Canadian palliative care literature.

***Design:*** A scoping review of medical databases was conducted to identify recent Canadian literature connecting culture and palliative care provision. This review yielded 21 relevant results from the past 10 years.

***Results:*** Ideas frequently mentioned in Canadian palliative care literature include cultural competency in health care providers, cultural sensitivity of treatment options, and cultural accessibility of available services. Issues that arose from the literature included differing ideas of the meanings of life and death, visibility of cultural minority groups, spiritual care needs, desire to involve friends and family in care, and misunderstandings of language and communication styles.

***Conclusion:*** The results of this review provide a starting point from which health care providers can begin lending attention to cultural determinants of health, thus improving palliative care services for diverse populations.

## Introduction

The Canadian Hospice Palliative Care Association^1^ states that good palliative care is holistic and gives attention to physical, spiritual, and psychosocial dimensions of health. The World Health Organization defines palliative care as “the prevention of suffering of…patients and their families facing the problems associated with life-threatening illness,”^2^ and similarly states that these problems include physical, psychological, social, and spiritual suffering.^3^ While attention to these factors is important, these statements are missing one critical emphasis: attention to cultural norms. Views of what constitutes a good life or good death are highly influenced by cultural values, perceptions, and beliefs.^4–8^ Consequentially, to provide holistic and effective palliative care, health care providers must be aware of the cultural norms affecting the spiritual and psychosocial needs of their patients and look for ways to facilitate culturally appropriate care for them.

Because Canada is an increasingly multicultural nation, it is critical that Canadian health care providers consider culture when creating individual care plans. To provide accessible and holistic care, health care providers must understand how culture influences palliative care needs and delivery in Canada.^4,7,8^ Unfortunately, few specific guidelines exist that could provide health professionals with a framework for understanding the influence of culture on care needs in Canada. Fang, Sixsmith, Sinclair, and Horst conducted a review in 2016 identifying systematic barriers and enablers that affect care for culturally and spiritually specific groups,^9^ however, it remains unclear what constitutes a “cultural” group in Canada, and questions remain regarding what specific interventions could be employed to improve care for culturally diverse groups. This review begins to fill this gap in the literature. The purpose of this review is to summarize how the concept of culture has been examined in Canadian palliative care research and synthesize insight on how health care providers can offer culturally relevant palliative care to patients.

## Methods

**FIG. 1.**
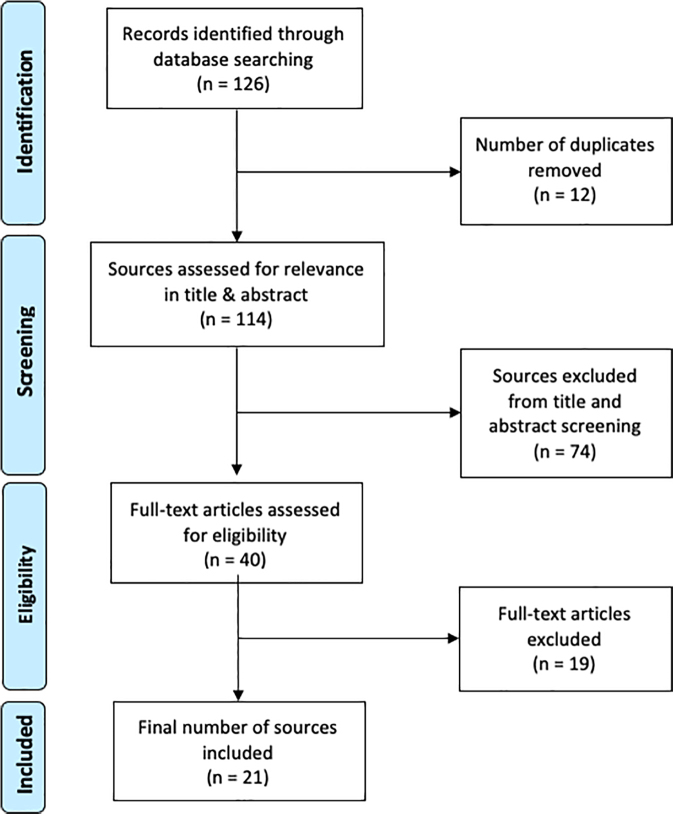
PRISMA^10^ record of the database search and screening process. Arrows indicate order of process. One hundred twenty-six sources were identified by the initial database search. Following removal of duplicates, 114 sources were screened for relevance to the research question in the title and abstract. Forty were screened for relevance in the full text. Twenty-one studies were identified for inclusion in this review.

A scoping review of medical databases was conducted to gather literature addressing culture in Canadian palliative care efforts (Fig. 1). Scoping reviews are useful for rapidly and systematically gathering literature pertinent to a highly specific research question and allow for the fast identification of broad gaps in the literature.^11^ Arksey and O'Malley present a scoping review framework that involves five steps: identifying the research question, gathering relevant studies, selecting studies for inclusion, charting of the data, and collating and summarizing data into a report.^11^ This study aimed to answer the question: How has patient culture been discussed in peer-reviewed Canadian palliative care literature in the past 10 years?

Relevant studies were gathered using the academic databases PubMed, Web of Science, and Embase. Gray literature was not reviewed so that the included sources were limited to peer-reviewed literature. An information specialist was consulted in the formation of the search strategy and assisted in the establishment of search terms. Sources were screened for study location and topic, as well as the inclusion of culture as a study concept. The keywords “Canada,” “Ontario,” “Manitoba,” “Saskatchewan,” “Alberta,” “British Columbia,” “Yukon,” “Northwest Territories,” “Nunavut,” “Newfoundland,” “Labrador,” “Quebec,” “New Brunswick,” “Nova Scotia,” and “Prince Edward Island” were used with the Boolean phrase “OR” to ensure that all research conducted in Canada was included in the initial search. The keyword “palliative care” was also used, with “end-of-life care” included in the search strategy as alternative phrasing as many researchers use the two terms synonymously. Finally, “culture” served as a last keyword to narrow literature to the scope of this review.

The initial search across all three databases yielded 126 potential sources. Studies for inclusion in the review were selected through two screening stages: one of the titles and abstracts of gathered studies, and one of the full texts. One reviewer screened the title and abstract of each source. Studies were included if they were (1) primary research articles, (2) contained the term “palliative care” or “end-of-life care” in the title or abstract, and (3) conducted in Canada between 2008 and 2018. This latter criterion was included to capture a recent picture (i.e., within 10 years of the review date) of how culture is discussed in palliative care literature, and to ensure that the discussion of terms used to refer to culture in care was relevant to current practice. Non-English studies were excluded. In this review, sources were not included or excluded based on source methodology.

Following removal of duplicates, one reviewer further assessed full-text articles for relevance to the research question. Articles were included if they explicitly discussed the role of patient culture in palliative or end-of-life care. Articles were excluded if their primary focus was “organizational culture”; this term refers to internal labor culture within care organizations rather than patient culture, and falls outside the scope of this article. This screening resulted in a final 21 articles for inclusion. Articles were charted according to the following categories: definitions or concepts of culture used, populations studied, and cultural issues raised. These categories provided a starting point for analysis, and themes emerged from the literature revealing how Canadian researchers, health care professionals, families, and communities think about culture in relation to palliative care. These themes are summarized in three sections: concepts used to discuss culture, populations in which culture is examined, and key elements of palliative care influenced by culture.

## Results

### Concepts used to discuss culture

Studies discussing culture in relation to palliative care needs used three common phrases and concepts related to culture: “cultural competency,” “cultural sensitivity,” and “cultural accessibility.” Closely examined, these concepts shed light on how Canadians think about culture as a phenomenon and provide insight about dominant perceptions and attitudes regarding its relevance within palliative care research. In this study, I provide a summary of how these ideas have been used in recent literature and how they relate to ideas about the provision of effective palliative care, and discuss the gaps, assumptions, implications, and limitations of these ideas.

#### Cultural competency

Studies referred to “cultural competency” as a necessity in the provision of effective palliative care to culturally diverse populations. When discussing this concept, Maddalena et al.^12^ reference Wells and Black's^13^ definition of cultural competency, defining it as “the process of actively developing and practicing appropriate, relevant, and sensitive strategies and skill in interacting with culturally different people” (p. 278). By this definition, cultural competency is a skill in constant development that contributes to a health provider's “capacity to respond to the needs of populations whose cultures are different from what might be called dominant or mainstream” (p. 278).^13^ Johnston et al.^14^ discuss cultural competency as a lead-in to cultural understanding. They argue that health care providers can gain cultural competence by reflecting on their own biases, emotions, and interests situated in their own cultures, allowing them to recognize when their own cultural norms influence their care decisions.^14^

Castleden et al.^15^ emphasize the need for training in cultural competency for health care providers. Jovanovic^16^ echoes this recommendation in her study of hospice volunteer experiences, suggesting that building cultural competency is a responsibility of hospice and palliative care providers. She suggests that content for trainings should begin with patients' cultural preferences. Understanding these preferences can generate the creation of training content.^16^ Additionally, Jovanovic^16^ suggests that maintaining cultural diversity among volunteer and staff travel experiences can contribute to an agency-wide level of cultural competency; if all cultural groups within the served area are represented by hospice volunteers and staff members, someone will always be available to offer culturally relevant care to patients. Additionally, exposing oneself to different cultures could allow opportunity to practice these skills.^16^

Finally, advocacy is a concept frequently discussed in relation to cultural competency. Johnston et al.^14^ suggest that health care providers who advocate for cultural competency among their staff create more accessible services for those who do not identify with the majority or mainstream culture. In recognizing distinct values, needs, and beliefs that exist within patients' cultural contexts, health care providers not only provide effective and quality care, but advocate for the human rights and validity of that cultural group.^14^

#### Cultural sensitivity

Canadian researchers discuss the concept of “cultural sensitivity” as a characteristic of good palliative care. Johnston et al.^14^ suggest that health care providers are responsible for creating a culturally sensitive care environment that positively affects patients and families. This environment is facilitated by intentional efforts to learn the beliefs and values of those accepting care.^14^ In contrast to cultural competency, which is often discussed as a learned skill in the literature, cultural sensitivity constitutes an attitude for professional practice involving a more nuanced awareness that individuals experience culture differently. By getting to know the person receiving care, health care providers may also get to know the patient's culture. This individualized approach, although requiring a great deal of listening and learning on the part of the provider, offers a comprehensive strategy for offering culturally sensitive care in palliative settings.^14^ Johnston et al.^14^ also extend this value to research, stating that studies exploring health care in specific cultural contexts must be carried out in a manner that is respectful of the culture's ways. They exemplify this principle in their study of the Mi'kmaq First Nation's palliative needs, valuing relationships within the research process, and beginning their exploration by gaining an understanding of Mi'kmaq historical and cultural context.^14^ Johnston et al.^14^ end their study with a call for inclusivity within the Canadian health care system, emphasizing the importance of including different worldviews in the planning, implementation, and design of health services to ensure culturally sensitive care for all residents of Canada.

#### Cultural accessibility

Within Canadian palliative care research, “cultural accessibility” is often referred to in tandem with a population's willingness to access services that are inconsistent with their cultural norms.^17–19^ In their study of palliative care in rural British Columbia, Castleden et al.^15^ found that health care providers perceived patients they defined as white and middle-class as more likely to access palliative care services, whereas Indigenous Peoples were considered more likely to “take care of their own” (p. 489).^15^ For these health care providers, the physical availability of care was considered equal for these groups, but they expressed that not all groups were equally interested in accessing this care. In light of this perception, Castleden et al.^15^ highlighted a need for a better understanding of Indigenous Peoples' needs and desires for palliative care, suggesting that they may be less likely to access care due to its lack of attention to cultural needs.

Similar results were found in Weerasinghe and Maddalena's^19^ study looking at palliative needs in South Asian immigrant populations in Halifax. This study found that South Asian families were less likely to access formal hospice or end-of-life care for their sick loved ones because of issues with clinical protocol.^19^ More specifically, South Asian families expressed concerns about (1) not being able to feed family members personally, a custom used to express love even when appetite is reduced in end-of-life circumstances, and (2) modesty with flimsy hospital gowns and discomfort with opposite-sex nursing.^19^ These concerns often led South Asian families to keep sick or dying loved ones at home without the assistance of formal palliative caregivers.^19^ Donovan and Williams^17^ observed a similar phenomenon in Vietnamese immigrant populations, reporting that Vietnamese families were likely to decline a given health care service if they considered it culturally inappropriate, citing that accepting care would be a waste and that it would be better offered to another family.

In their study of rural palliative care needs, Pesut et al.^18^ found that the feeling of “being known” was important for rural community members when accessing care. Palliative patients were less likely to access home or hospice care in rural communities if they knew or thought they would be cared for by strangers.^18^ Likewise, participants in Pesut et al.'s study noted that prominent community members were more likely to access and be satisfied with palliative care because caregivers were more likely to know them and give personal attention. This led to an inequity in care quality; those who were leaders or held power in the community received what they perceived to be better care.^18^ To rebalance this inequity, McKee et al.^20^ suggest the employment of community volunteers to act as “cultural navigators” (p. 109). They suggest that these volunteers might know the community in different ways from those providing care and help bridge cultural disparities to provide more personal care.^20^

### Populations in which “culture” is examined

While cultural palliative needs have been studied within several distinct groups in Canada (Table 1), it remains unclear following a review of the literature what groups are considered “culturally distinct” and on what bases distinctions are made. Still, an examination of which groups have been the focus of studies of culture and palliative care in the Canadian context offers a starting point for clarifying how cultural difference is defined and applied in the Canadian context.

**Table 1.** Summary Chart of Articles Included in This Review

Population	Author(s)	Year	Themes discussed				
			Meanings of life and death	Visibility	Spiritual/religious care	Family/community involvement in care	Language
Indigenous groups							
Rural Ontario town with Ojibway & Cree aboriginal residents	Kelly, Linkewich, Cromarty, St. Pierre-Hansen, et al.	2009					X
Aboriginal families in British Columbia	Castleden, Crooks, Hanlon and Schuurman	2010	X	X	X		
Aboriginal Elders in southern Saskatchewan	Hampton, Baydala, Bourassa, McKay-McNabb, et al.	2010	X		X		
Mi'kmaq	Johnston, Vukic, and Parker	2013	X		X	X	X
Inuit	Hordyk, Macdonald and Brassard	2017					X
Naotkamegwanning First Nation	Nadin, Crow, Prince and Kelley	2018	X				
Six Nations of the Grand River; Naotkamegwann-ing, Fort William, and Peguis First Nations	Kelley, Prince, Nadin, Brazil, et al.	2018	X		X	X	
Other ethnic groups							
African Canadians	Maddalena, Bernard, Etowa, Murdoch, et al.	2010		X			
	Maddalena, Bernard, Davis-Murdoch and Smith	2013		X		X	
Chinese Canadians	Seto-Nielson, Angus, Gastaldo, Howell, et al.	2013	X				
Vietnamese Canadians	Donovan and Williams	2015			X	X	
South Asian Immigrants	Weerasinghe and Maddalena	2016			X		
Religious groups							
Sikh	Ebrahim, Bance and Bowman	2011			X	X	
Dutch-reformed	Donovan, Williams, Stajduhar, Brazil, et al.	2011			X		
Rural communities							
Northwestern Ontario	McKee, Kelley, Gulrguis-Younger, MacLean, et al.	2010			X		
Quebec	Veillette, Fillion, Wilson, Thomas, et al.	2010				X	
Western Canada	Pesut, Bottorf, and Robinson	2011	X			X	
Western Canada	Pesut, Robinson and Bottorff	2014				X	
Palliative care providers							
Alberta	Sinclair	2011	X				
Across Canada	Giesbrecht, Crooks, Williams and Hankivsky	2012			X		
Greater Toronto area	Jovanovic	2012					

Eight out of 21 palliative care studies that included culture in their discussion were conducted in Indigenous populations.^14,15,21–25^ Castleden et al.^15^ justifies the study of cultural palliative needs in Indigenous communities in their research on palliative care in rural British Columbia, emphasizing that, to strengthen palliative care approaches for Indigenous patients, health care providers must acknowledge the cultural, linguistic, and ethnic diversity that exists among Indigenous communities. Each people group has its own set of customs, values, and beliefs, and should be treated and studied as a distinct cultural group.^15^

Six of the reviewed studies focus on non-Indigenous populations. These populations include groups that are labeled in the Canadian Census and also often define themselves as culturally or ethnically distinct, and include Chinese Canadians,^26^ Vietnamese Canadian,^17^ and South Asian immigrants,^19^ as well as groups identified as culturally distinct based on racialized identities, such as African Canadians.^12,27^ The exploration of culturally specific values and preferences for end-of-life palliative care have also been explored with a focus on religiously defined groups, including Sikh^28^ and Dutch Reformed populations in Canada.^29^

Five of the studies included in this review address culture in relation to rural palliative needs.^16,18,20,30,31^ Studies also identify rural culture as distinct from urban culture,^15,31^ specifying that urban models of care cannot be transplanted into rural contexts because perceptions of good care are influenced by rural residents' lived experiences.^31^ These experiences are shaped by political, social, and economic realities that exist within rural contexts.^31^ Rural individuals believed themselves to have unique perspectives about what constituted a “good death,” and held a deep commitment to their communities that was not as prevalent in urban areas.^31^ In contrast, Veillette et al.^30^ warn against the homogenization of “rural” Canada as one distinct culture in itself. Pesut et al.^31^ found that participants in a study on rural palliative care needs considered their communities culturally separate from surrounding rural communities and were quick to distinguish themselves from neighboring towns as a unique context. To account for this phenomenon and avoid the homogenization of culture to all rural communities, Veillette et al.^30^ examined culture in two different rural regions in Quebec. Veillette et al.^30^ is the only study in this review to make a cultural distinction between rural communities.

Some researchers approach the study of culture at a broad level, such as Pesut et al.'s^31^ study of rural palliative needs; while others address specific ethnocultural, racial, linguistic, or religious groups, such as Ebrahim et al.'s^28^ study of Sikh preferences or Donovan and William's^17^ study of Vietnamese Canadian palliative care needs. Three studies did not study culture in any particular group, but instead examined it generally as a concept from the perspectives of palliative care providers.^16,32,33^ The defining and locating of culture in such diverse ways indicates potential assumptions as well as challenges of studying culture and palliative care in tandem.

### Key elements of palliative care influenced by culture

Having reviewed the ways culture has been discussed in Canadian palliative care literature, we can now look at what elements commonly arise within these discussions (Table 1). This section provides an overview of key elements related to the provision of palliative care through the lens of culture. These key considerations provide a starting point for exploring intersections of palliative care needs and cultural norms in specific settings.

#### Meanings of life and death

Several studies cite differences in views of life and death as barriers to culturally appropriate care.^14,15,18,21,26^ Hampton et al.'s^21^ study interviewing elders of Indigenous communities found that elders want palliative care providers to understand the meaning of death within an Indigenous context. Elders stated that in their view, life and death were entwined—that death was not a symbol of the end of life, but a part of life itself, “as necessary as birth” (p. 9).^21^ Elders expressed concern over the constant competition that exists between Western and Indigenous models of care.^21^ In the Western biomedical context, death is considered the enemy and experienced in a technical manner. From at least some Indigenous perspectives, however, death represents a transition from one life to the next; it is viewed as a journey to the Spirit World.^21^ Elders in Hampton et al.'s^21^ study expressed wishes that palliative care providers would recognize and respect their Indigenous view of death and create care procedures that were consistent with this view and assist the process of “dying healed” (p. 7). To ensure this, Nadin and colleagues^20^ call for partnerships between researchers, health care providers, and Indigenous communities to determine cultural norms relating to life and death, and to incorporate these into palliative care programs.

Castleden et al.^15^ discuss experiences of life and death as cultural phenomena that are entwined with cultural ideas of what constitutes a “good life” or “good death.” The Western idea of a “good death” is often viewed as one that is resisted; in the biomedical view, “fighting” illness is viewed as admirable, and combative language is often employed when speaking of illness (e.g., a “battle” with cancer).^30^ Sinclair^32^ also explored the concept of a “good death” in palliative care professionals and found that two views of a “good death” existed: an integrated view, in which death was considered a “continuum of life,” (p. 182) and a disintegrated view, in which death is considered unnatural and in discord with a person's hopes for his or her life. The view of death that a person holds is highly contingent on cultural context.^32^ As such, a person's culture can be telling of his or her view and provide health professionals with insight into his or her state of mind as death approaches.

#### Visibility

The concept of visibility was commonly discussed in Canadian palliative literature, with particular emphasis on the *invisibility* of minority cultures. Castleden et al.'s^15^ interviews with British Columbian palliative health professionals revealed that outsiders considered the Sinixt Indigenous Peoples extinct, even though they still existed in small numbers. This perception was held largely, according to Castleden et al.,^15^ because health professionals had never treated Sinixt patients before. The authors posited that this may have been a reality because many Sinixt people not seeking palliative services due to cultural differences.^15^ Invisibility can also occur because of stereotypical views of what an Indigenous person “should” look like. Interviewed health care providers indicated that when an Indigenous individual does not match a stereotypical idea of what non-Indigenous health care providers think they “should” look like, they have difficulty identifying them.^15^ Conversely, in cases where Indigenous individuals met health care providers' stereotypical view of what an Indigenous person “should” look like, interviewed providers offered treatment according to their assumptions about what Indigenous patients wanted, rather than asking.^15^ Because of this challenge with discernment, there is heavy reliance on Indigenous Peoples to self-identify to access the care they need.^33^ It is clear from Castleden et al.'s^15^ study that Indigenous People remain mysterious to many non-Indigenous health professionals, further alienating them as a care-seeking population and contributing to their invisibility.

Maddalena et al.^12^ found results similar in Nova Scotia's African Canadian population. Maddalena et al.^12^ found that Nova Scotian African Canadian families preferred to care for their sick or dying loved ones at home rather than enroll them in a hospice, rendering them an invisible population to health care providers. Later research by Maddalena et al.^27^ demonstrated that palliative services were of interest to African Canadian families across the Atlantic provinces, however, services that were of most interest supported their cultural desire to care for loved ones at home. Maddalena et al.^27^ conclude their study with an emphasis on increasing community awareness in a culturally sensitive manner, in this case the holding of a town hall meeting to communicate available services. By exercising intentional efforts to communicate with cultural populations, culturally relevant services become more accessible.^27^

#### Spiritual and religious care

Several studies looking at palliative needs in specific cultural contexts expressed the need for adequate spiritual and psychosocial services. In many instances, spiritual needs mapped to religious needs. In Ebrahim et al.'s^28^ study of Sikh end-of-life preferences, it was expressed that prayer, recitations, and hymns played large roles in family bereavement. Ebrahim et al.^28^ recommended that, for hospitals to offer culturally sensitive care, Sikh families must be allowed to perform these religious or spiritual practices in close proximity to the patient.

Weerasinghe and Maddalena's^19^ study of South Asian end-of-life needs found that religious beliefs were entwined with perspectives of death. Examples of these needs included the Buddhist belief in rebirth—that death signified the end of suffering and a new beginning—and the Hindu belief that a family member's good Karma might provide hope for recovery.^19^ Weerasinghe and Maddalena^19^ echo Ebrahim et al.'s^28^ call that Canadian hospitals must be accommodating to religious rituals and practices to care for the spiritual wellbeing of patients and their families.

One study examining palliative needs in Nova Scotian African Canadian communities identified that sense of wellbeing was linked to prayer, Bible reading, and the involvement of church in palliative care.^20^ In one case, a patient wanted to be baptized by immersion while receiving palliative care in preparation to be with God.^20^ Throughout Maddalena et al.'s^20^ study, the concept of “fatalism,” or the idea that one's fate is in the hands of God, was prevalent.

For Vietnamese immigrants, caring for spiritual health of family members at the end of life meant telling happy stories from the past and engaging in prayer.^17^ In Dutch Reformed communities, religion was labeled as a critical element of cultural identity that affected every care decision.^29^

The literature search surfaced several articles that included reference to preparation for the afterlife in Indigenous communities in Canada.^14,15,21,33^ The desire to practice prayer, ceremonial sweats, and gathering of loved ones to “let go” of the patient indicate that spirituality and religion played a substantial role in palliative care for many Indigenous residents of Canada and must be given attention by health care providers.^14,15,21^ The results of these studies imply that the Canadian understanding of spirituality as it relates to culture often maps to religious care in a palliative care context.

#### Family and community involvement in caregiving

Studies examining palliative care in cultural minorities regularly cited family involvement in care and care decisions as a significant issue in palliative care provision. Among the Mi'kmaq, Johnston et al.^14^ found that family members desired to perform personal tasks for their sick loved ones, including laundry, cooking meals, and housekeeping, as an indication of love and support. Family and friends also played a large role in that patient's journey to the Spirit World. It was culturally normal for many people to gather with the patient while they were in the hospital so that they would not have to make the journey alone.^14^

Among Sikh patients, family was considered the primary health care decision maker.^28^ In Sikh families, the eldest son held responsibility for end-of-life decisions for his parents, and it was considered respectful for health care providers to consult the family before delivering news of prognosis.^28^ Similar responsibility was observed in Vietnamese families; however, the primary caregiving role traditionally fell to the wife of the eldest son and was almost exclusive to parental care for immediate family.^17^

Among Atlantic African Canadian families, Maddalena et al.^27^ found that home caregiving was the most common form of end-of-life care, and that caregivers considered it a personal sacrifice and the ultimate act of love to care for sick or dying loved ones. Similar results were gathered by Pesut et al.^31^ and Veillette et al.^30^ who found that rural palliative patients wanted to die surrounded by family and friends, and that cultural meaning was associated with family providing informal home palliative care. In rural communities, a strong emphasis exists on the values of community and mutuality.^18^ Neighbors are considered “necessary,” and hold a role in providing emotional support for palliative patients.^18^ These studies point to the importance of including family and community in the care of palliative patients, and that in doing so, cultural needs may be met within care.

#### Language and communication

Three studies reported language as important to the provision of culturally relevant care. All studies mentioning the centrality of language and choice of phrasing when providing care to culturally relevant care were conducted in Indigenous populations. For example, Johnston et al.^14^ reported the Mi'kmaq preference not to use the term “end-of-life” when discussing death because the Mi'kmaq viewed death as a continuation into the next life. Consequentially, referring to care as “end-of-life” care was culturally inappropriate. A study by Hordyk et al.^23^ exploring Inuit translator experiences with palliative care further explored this idea of culturally appropriate language. Hordyk et al.^23^ found that interpreters were usually relied on to transmit news of death. One interpreter expressed that interpreters should “never interpret word for word” (p. 5) as the way non-Inuit doctors phrased the news was insensitive.^23^

In addition to the importance of providers understanding culturally specific terms and meanings of words when working with Indigenous populations, manner of communication was also explored in culture-related palliative care literature. Kelly et al.^24^ found that Cree and Ojibway Peoples in Northwestern Ontario preferred direct communication from doctors and expressed that they did not want to be given false hope. Furthermore, interviewed Indigenous individuals reported wanting this communication to include words of encouragement, however, there was no specification of what this communication could sound like.^24^ It should be noted that these insights arose from specific groups and in the context of relatively small studies, these findings may not be generalizable across diverse Indigenous groups in Canada.

## Discussion

### Implications of culture-related language on palliative care provision

In the above-summarized results, “cultural competency,” “cultural sensitivity,” and “cultural accessibility” arose as common terms used in Canadian palliative care literature. Considering the contexts in which these terms are used in Canadian palliative care literature, some assumptions and implications of how they are distinguished from one another arise.

In the manner presented by the literature, “cultural competency” appears to refer to a learnable, and perhaps teachable, skill that health care providers should aim to gain to benefit their practice. This finding exemplifies a problematic potential slippage between acknowledging the importance of being self-aware, encouraging sensitivity to and respect of unfamiliar norms, and claiming cultures are somewhat easily learnable and static.^34^ While recognizing that calls for cultural competency are well intended, Botelho and Lima^34^ caution against use of this term in ways that are overly simplistic and can ultimately undermine the good intentions. Although seeking to become more culturally competent can improve health care providers' responsiveness to culture in practice, it can also perpetuate assumptions about cultural experience and reduce patients to their cultural backgrounds. It can also obscure limitations in crosscultural understanding; health care providers cannot expect mastery over a culture that is not their own. Botelho and Lima^34^ further note that if seeking to build cultural competency, health care providers should not lose sight of the potential blind spots that can accompany use of the term. They should also not lose sight of the individual factor in treating patients, and acknowledge cultural aspects of care alongside other social, historical, and personal factors.^34^

There is an explicit awareness of culture as one of several dimensions influencing patient's needs present in the literature on cultural sensitivity in palliative care. In the reviewed studies, cultural sensitivity refers to an awareness of culture that underlies practice and acknowledges the individual living in relation to the culture. Finally, “cultural accessibility” acts as a descriptor for a system of care; is the care offered within this system accessible in its practice to the cultural groups it aims to serve? Studies suggest that systems are only culturally accessible to the groups for which it was directly designed. In this sense, the Canadian biomedical system built to meet the needs of the Western norm may not be reasonably transferable to cultural minorities who do not view health or illness in a Western way. Building culturally accessible services, then, requires a back-to-basics approach that will examine cultural norms and design practice to meet needs influenced by those norms.

### What constitutes a “cultural” population in Canada?

Canadian palliative care research examining care needs through a cultural lens is not extensive. Still, it is clear from the reviewed studies that knowledge gained from such research has begun to generate valuable and nuanced insights into diverse, population-specific preferences, expectations, and beliefs associated with palliative care. The variety of populations targeted by this research reveals that “culture” is a recurring term for referencing less clinical, more social, and psychosocial patient needs. Also important to emphasize is that in Canadian palliative care literature, “cultural” factors include religious, ethnic, racial, and geographical factors that interact with one another to form a complex mosaic of lived experience that sets the individual apart from the “majority.” Interestingly, this “majority” is never explicitly mentioned in the literature, but implicitly refers to a population in which an absence of culturally specific needs exist. Culturally relevant, sensitive, and accessible care, if we follow the lead of this literature, is important to the provision of high-quality care for particular individuals considered “other/cultural” when compared with the implied “culture-free” population of non-Indigenous, nonracialized, white, urban-residing individuals. Whether a group is “cultural” is therefore based on either their Indigenous, ethnic, or racialized identity, rural location, or religious affiliation.

This finding draws attention to a major pitfall in the Canadian understanding of culture; while cultural difference is usually used to define practices and values of demographic minorities, all social actors in a given national context harbor culture. It is a person's position in society, as a member of a dominant or nondominant cultural group that facilitates the framing of their practices and values as “cultural,” where “cultural” could be equated to “different” versus “normal.”^35^

Many authors,^12,14,17,19^ consistent with social anthropological theory, define culture as a dynamic force that exists at many levels and disperses through populations in complex ways. For example, while a distinguishable culture may encompass Canada, another distinct culture might exist that is specific to rural Canada. Furthermore, rural Canada might display multiple cultures specific to communities within it. To layer the concept further, within each rural community there may exist additional subcultures, such as specific social, racialized, and religious groups. This layering concept continues at an infinite level, which can make attending to culture in palliative care a difficult and overwhelming task.

This complexity raises the question: How can the concept of culture be adequately examined in the contexts of Canadian palliative care? One way researchers might approach this question is through the lens of Leininger's^36^ Culture Care Theory. Based on anthropological principles, Culture Care Theory postulates that cultural factors affect the health needs of *all* people, not only those who identify with cultural minorities.^36^ It proposes that similarities and diversities exist between cultures; that worldview, cultural, and social factors influence care outcomes; and that both cultural and professional factors influence how health professionals offer care.^36^ When examined together, these aspects of Culture Care Theory provide a rough framework from which Canadian researchers can explore cultural palliative needs. By recognizing the unique cultural context of each population studied, health care providers can develop specific strategies to offer culturally relevant and accessible care for palliative patients.^37^

### Limitations and future directions

By nature of the scoping review methodology, this review gathered a highly specific and recent overview of relevant literature. A more exhaustive systematic review could capture *more* relevant literature, including both gray literature and academic sources that present culture as a care consideration, but do not explicitly focus on it. Future reviews could also expand the publication timeframe to observe how the language and thinking concerning culture in palliative care has evolved over time.

## Conclusion

It is clear from this review that culture plays a large role in assuring effective and holistic palliative care for a number of Canadian populations. It has been shown that what constitutes a norm for one community does not directly translate to another. There is a demonstrated need for cultural competency and sensitivity on the parts of Canadian health care providers, particularly those working in urban areas where cultural differences are more likely to be observed. By being reflexive about what we mean by “culture” in a palliative care context, lending attention to common cultural norms related to views of life, illness and death, spiritual and psychosocial needs, involvement of family and community in care, as well as holding an awareness of visibility (or invisibility) of cultural minorities, health care providers can create culturally accessible palliative programs. The continued study of values and preferences that are central to defining good care in the context of one's experience of culture will contribute to a more holistic health system equipped to care for *all* residents of Canada, not only those identifying with the cultural majority.
